# Hyperprogression after immunotherapy in patients with malignant tumors of digestive system

**DOI:** 10.1186/s12885-019-5921-9

**Published:** 2019-07-17

**Authors:** Zhi Ji, Zhi Peng, Jifang Gong, Xiaotian Zhang, Jian Li, Ming Lu, Zhihao Lu, Lin Shen

**Affiliations:** Department of Gastrointestinal Oncology, Key Laboratory of Carcinogenesis and Translational Research (Ministry of Education), Peking University Cancer Hospital & Institute, Fucheng Road 52, Haidian District, Beijing, 100142 China

**Keywords:** Hyperprogression, Immunotherapy, Digestive system, Tumor growth kinetics (TGK), irRECIST

## Abstract

**Background:**

Immune checkpoint inhibitors (ICIs) were approved to have a significant antitumor activity in various tumor types. In practice, some patients do not seem to benefit from ICIs but rather to have accelerating disease. The aim of this study was to evaluate hyperprogression in patients with malignant tumors of digestive system treated with ICIs.

**Methods:**

Medical records from consecutive patients with malignant tumors of digestive system treated with ICIs in Peking University Cancer Hospital were retrospectively collected. Tumor growth kinetics (TGK) on immunotherapy and TGK pre-immunotherapy were collected and TGK ratio (TGKR) was calculated. Hyperprogression was defined as TGKR≥2.

**Results:**

From August 2016 to May 2017, 25 evaluable patients were identified from 45 patients with malignant tumors of digestive system. Five patients were considered as having hyperprogression. Three of 5 were neuroendocrine carcinomas (NECs) and the other 2 were adenocarcinomas. Four of 5 were treated with programmed cell death ligand 1 (PD-L1) inhibitor, the other one was treated with PD-L1 inhibitor combined with cytotoxic T lymphocyte associated antigen-4 (CTLA-4) inhibitor. Pseudoprogression was observed in 2 patients.

**Conclusions:**

Hyperprogression was observed in a fraction of patients with malignant tumors of digestive system treated with ICIs. Further investigation is urgently needed.

## Background

Immunotherapy has become a new method to refractory or recurrent tumors. A number of clinical studies have confirmed that immune checkpoint inhibitors (ICIs) had a significant antitumor activity in various tumor types [1–5]. The new immunotherapy also results in novel tumor response patterns such as delayed tumor response or pseudoprogression [6, 7]. What’s more, researchers found that ICIs might have a deleterious effect by accelerating the disease in a subset of patients which was described as “hyperprogressive disease” or “hyperprogression” [8–10].

Champiat et al. [8] reported occurrences of rapid progression on ICIs and described as “hyperprogressive disease” for the first time. Hyperprogression was defined as a RECIST progression at the first evaluation and as a ≥ 2-fold increase of the tumor growth rate (TGR) compared with pre-immunotherapy. Nine percent (12/131) of evaluable patients were considered as having hyperprogression. Kato et al. [9] observed same phenomenon and attempted to explore the genetic markers associated with hyperprogression. Time to treatment failure (TTF) < 2 months, > 50% increase in tumor burden and > 2-fold increase in progression pace (PP) were considered as hyperprogression. Saada-Bouzid et al. [10] investigated hyperprogression in recurrent and/or metastatic head and neck squamous cell carcinoma (R/M HNSCC) patients. Hyperprogression was defined as ≥2-fold increase of the tumor growth kinetics (TGK) compared with pre-immunotherapy. Hyperprogression was observed in 29% (10/34) patients.

We also recently identified a subset of patients with malignant tumors of digestive system whose disease paradoxically accelerated on immunotherapy. Herein, we describe our patients of hyperprogressors and discuss the related questions to hyperprogression.

## Methods

### Patients

Medical records from consecutive patients with malignant tumors of digestive system enrolled and treated in phase I clinical trials with programmed cell death-1/ programmed cell death ligand 1 (PD-1/PD-L1) inhibitor alone or combined with cytotoxic T lymphocyte associated antigen-4 (CTLA-4) inhibitor in Peking University Cancer Hospital between August 2016 and May 2017 were retrospectively collected (NCT02825940, NCT02978482, NCT02915432, NCT03167853, CTR20160872). All patients had histologically confirmed malignant tumors of digestive system.

### Assessments

T_PRE_, T_0_, and T_POST_ stand for the time of pre-baseline, baseline, and first evaluation imaging, respectively. S_PRE_, S_0_, S_POST_ stand for the tumor burden (irRECIST) at pre-baseline, baseline, and first evaluation imaging, respectively. So the measurable new lesions will be added into the total tumor burden. Besides, if there is no target lesion at pre-baseline, target lesions chosen at baseline will be retrospectively analyzed at pre-baseline. The pre-baseline TGK (TGK_PRE_) was defined as the difference of the tumor burden per unit of time between pre-baseline and baseline imaging: (S_0_-S_PRE_)/(T_0_-T_PRE_). Similarly, the post-baseline TGK (TGK_POST_) was defined as (S_POST_-S_0_)/(T_POST_-T_0_). The TGK ratio (TGKR) was defined as the ratio of TGK_POST_ to TGK_PRE_. Hyperprogression was defined as TGKR≥2.

For the categorical variable data χ^2^ or Fisher’s exact test was used and for the numerical variable data t test or Mann-Whitney test was used. Data input and statistical analysis were performed using SPSS 21.0 statistical software. The significance test was a two-sided test and *P* < 0.05 considered statistically significant differences.

## Results

**Fig. 1:**
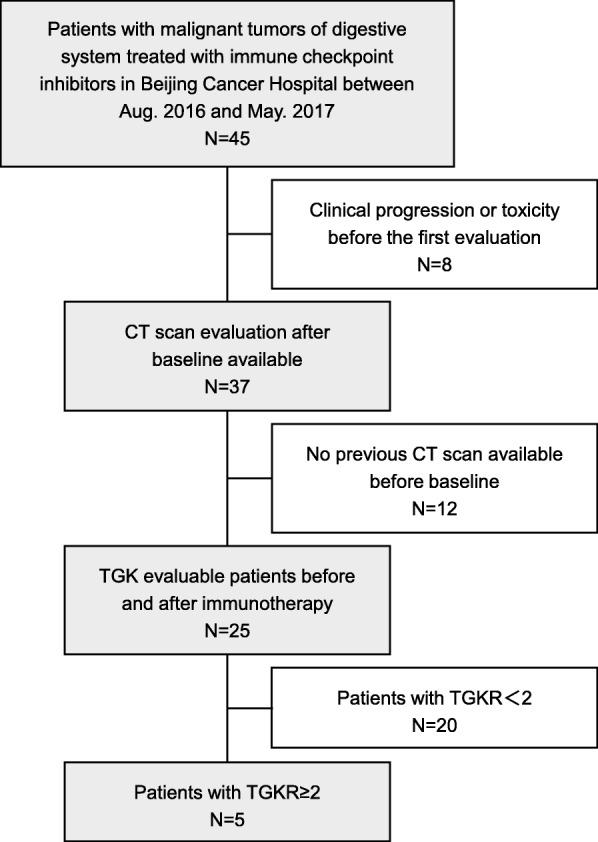
Flowchart of study selection process

We analyzed a total of 45 patients with malignant tumors of digestive system who enrolled and treated in phase I clinical trials with PD-1/PD-L1 inhibitor (alone or combined with CTLA-4 inhibitor) in Peking University Cancer Hospital between August 2016 and May 2017. All of them had the baseline CT scans. As illustrated in the flowchart (Fig. 1), a total of 8 patients (18%) terminated treatment because of clinical progression or toxicity before the first tumor evaluation. Of the other patients, 12 patients (27%) did not have a previous CT scan available before baseline. Then 25 patients (56%) could be explored for TGK_PRE_ and TGK_POST_.

Patient characteristics are described in Table 1. Median age was 54 years. Primary tumor locations were stomach, esophagus, colorectal, liver, pancreas and ampulla in 8 (32%), 7 (28%), 7 (28%), 1 (4%), 1 (4%) and 1 (4%) patients, respectively.

**Table 1:** Patient characteristics

	All patients (*n* = 25)	TGKR<2 (*n* = 20)	TGKR≥2 (*n* = 5)	*P* value
Gender				
Male	17 (68%)	14 (70%)	3 (60%)	1.000
Female	8 (32%)	6 (30%)	2 (40%)	
Age	54 (22–77)	52 (22–77)	63 (31–65)	0.587
EGOG				
0	14 (56%)	10 (50%)	4 (80%)	0.341
1	11 (44%)	10 (50%)	1 (20%)	
Location				
stomach	8 (32%)	6 (30%)	2 (40%)	1.000
esophagus	7 (28%)	6 (30%)	1 (20%)	
colorectal	7 (28%)	5 (25%)	2 (40%)	
liver	1 (4%)	1 (5%)	0	
ancreas	1 (4%)	1 (5%)	0	
ampulla	1 (4%)	1 (5%)	0	
Histology				
adenocarcinoma	14 (56%)	12 (60%)	2 (40%)	0.032
squamous carcinoma	6 (24%)	6 (30%)	0	
neuroendocrine carcinoma	4 (16%)	1 (5%)	3 (60%)	
hepatocellular carcinoma	1 (4%)	1 (5%)	0	
Metastatic site				
≤2	16 (64%)	13 (65%)	3 (60%)	0.749
>2	9 (36%)	7 (35%)	2 (40%)	
Type of immunotherapy				
PD-1 inhibitor	6 (24%)	6 (30%)	0	0.447
PD-L1 inhibitor	16 (64%)	12 (60%)	4 (80%)	
PD-L1 + CTLA-4 inhibitor	3 (12%)	2 (10%)	1 (20%)	
MMR				
pMMR	6 (40%)	4 (33%)	2 (66.7%)	0.525
dMMR	9 (60%)	8 (67%)	1 (33.3%)	
PD-L1				
positive	6 (42%)	5 (46%)	1 (50%)	1.000
negative	7 (54%)	6 (55%)	1 (50%)	

PD-L1 positive meant combined positive score ≥ 1% and PD-L1 negative meant combined positive score < 1%. Abbreviation: *MMR* mismatch repair, *pMMR* mismatch repair proficient, *dMMR* mismatch repair deficient

**Fig. 2:**
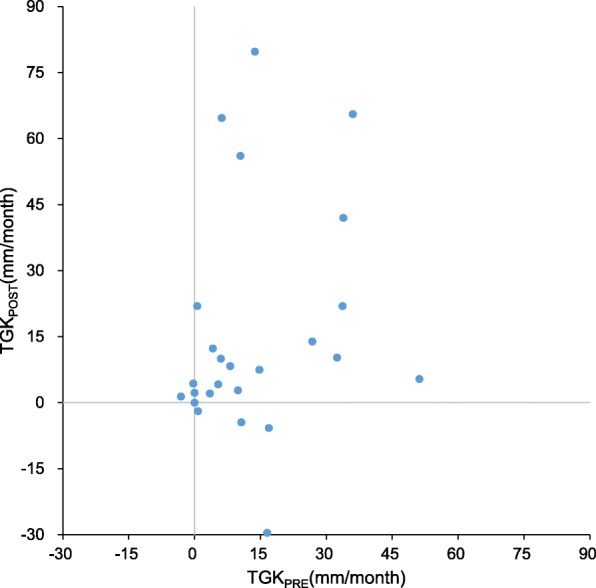
Pairwise comparisons of TGK on immunotherapy (TGK_POST_) and TGK pre-immunotherapy (TGK_PRE_) in 25 patients with malignant tumors of digestive system enrolled and treated in phase I clinical trials with ICIs. Each dot represents a patient

By irRECIST, a total of 15 (60%), 8 (32%) and 2 (8%) patients exhibited progressive disease (PD), stable disease (SD) and partial response (PR), respectively. The distribution of TGK on immunotherapy and TGK pre-immunotherapy are shown in Fig. 2. Patients with TGK_POST_>0 meant that tumor growth and TGK_POST_<0 meant tumor shrinkage. And the slope connect the dot and original point indicated the tumor growth rate, TGK_POST_/TGK_PRE_>1 meant tumor growth acceleration and TGK_POST_/TGK_PRE_<1 meant tumor growth deceleration. Hyperprogression was observed in 5 patients. Three of 5 were neuroendocrine carcinomas (NECs) and the other 2 were adenocarcinomas. Four of 5 were treated with PD-L1 inhibitor, the other one was treated with PD-L1 inhibitor combined with CTLA-4 inhibitor.

Pseudoprogression was observed in 2 patients with colon carcinomas. The first evaluation after immunotherapy showed PD with TGKR of 1.67 and 0.11 respectively but the general condition was improved which encouraged continued immunotherapy. The second evaluation of the 2 patients both showed SD which confirmed the pseudoprogression.

## Case reports

**Fig. 3:**
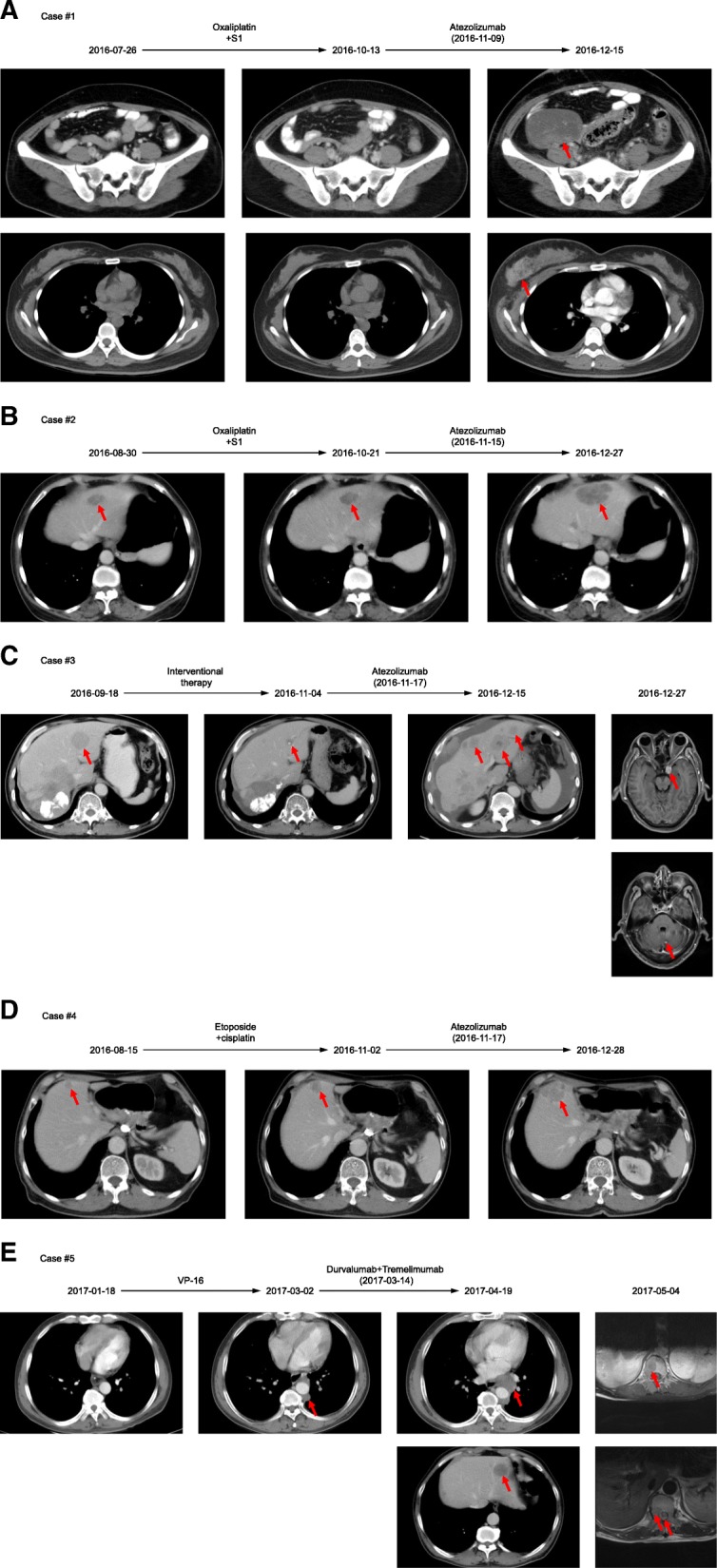
Serial imaging before and after immunotherapy in the five hyperprogressors. Pre-baseline imaging refers to images about 2 months before immunotherapy. Baseline imaging refers to imaging immediately before immunotherapy. **a**, Case #1: patient with right colon signet-ring cell carcinoma. Restaging imaging done 1.2 months after starting atezolizumab showed multiple new metastases including right breast, bilateral ovaries et al. (863% increase from baseline imaging). Patient died 3.6 months from starting atezolizumab. **b**, Case #2: patient with gastric adenocarcinoma. The first evaluation done 1.4 months after the initiation of atezolizumab revealed a rapid progression of liver masses as well as new liver metastasis (107% increase from baseline imaging). Patient subsequently received liver interventional therapy and died 7.4 months from the initiation of atezolizumab. **c**, Case #3: patient with colon NEC. After two cycle’s atezolizumab therapy, patient presented severe abdominal distension, scans (0.94 months post atezolizumab) showed rapid progression of the peritoneum and liver metastases and new brain and adrenal gland metastases (139% increase from baseline imaging). Patient died 2.1 months from starting atezolizumab. **d**, Case #4: patient with gastric NEC. CT scans (1.4 months post atezolizumab) revealed a 44% increase in the liver mass and died 5.6 months from the initiation of atezolizumab. **e**, Case #5: patient with esophagus NEC. Patient had an incomplete bowel obstruction after immunotherapy and scans (1.2 months post durvalumab and tremelimumab) showed new lung, liver, T10–12 and L2–4 metastases (538% increase from baseline imaging). He died 3.8 months from starting durvalumab and tremelimumab

**Fig. 4:**
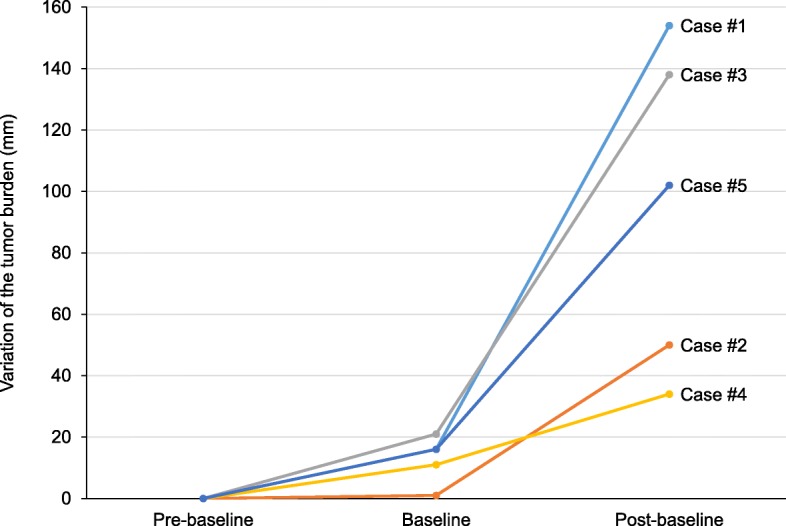
Variation of the tumor burden in the five hyperprogressors. Tumor burden is compared from about 2 months before immunotherapy (pre-baseline) to image immediately before immunotherapy (baseline), and then to first imaging after immunotherapy (post-baseline). Tumor burden was evaluated with irRECIST

We describe all 5 patients with malignant tumors of digestive system treated with ICIs who were considered as hyperprogressors. Serial imaging before and after immunotherapy in the five hyperprogressors are shown in Fig. 3 and variation of the tumor burden are shown in Fig. 4.

### Case #1

A 31-year-old woman with right colon signet-ring cell carcinoma metastatic to the peritoneum was started on the PD-L1 inhibitor atezolizumab. Immunohistochemistry showed that microsatellite instability-high (MSI-H) and PD-L1 (−). Prior therapies included radical operation for right colon carcinoma followed by S1/oxaliplatin. Restaging imaging done 1.2 months after starting atezolizumab showed multiple new metastases including right breast, bilateral ovaries, T3, T9, T12, L2 and lymphadenopathy (863% increase from baseline imaging) (Fig. 3a). Superficial lymph node biopsy at the time of progression did not reveal signs of pseudoprogression, including lymphocyte infiltration or tumor necrosis. Patient’s performance status fell sharply and died 3.6 months from starting atezolizumab.

### Case #2

A 63-year-old woman with Her-2 positive gastric adenocarcinoma showed slow progressive liver, lung and peritoneum metastases while sequentially received trastuzumab/capetacibine/oxaliplatin, paclitaxel/capetacibine, and pyrithione et al. The first evaluation done 1.4 months after the initiation of atezolizumab revealed a rapid progression of liver masses as well as new liver metastasis (107% increase from baseline imaging) (Fig. 3b). Liver mass biopsy afterwards excluded the possibility of pseudoprogression. Patient subsequently received liver interventional therapy and died 7.4 months from the initiation of atezolizumab.

### Case #3

A 63-year-old man with colon NEC metastatic to lung, liver, spleen, peritoneum and lymph nodes had palliative surgery followed by first-line therapy with etoposide/cisplatin. Afterwards liver interventional therapy was done but the efficacy was limited. After two cycle’s atezolizumab therapy, patient presented severe abdominal distension, which prompted the physician to obtain CT imaging. Scans (0.94 months post atezolizumab) showed rapid progression of the peritoneum and liver metastases as well as new brain and adrenal gland metastases (139% increase from baseline imaging) (Fig. 3c). Patient received radiotherapy to the brain metastases but died 2.1 months from starting atezolizumab.

### Case #4

A 65-year-old man with gastric NEC metastatic to liver received radical operation for gastric cancer and adjuvant therapy with etoposide and cisplatin. Therapy was switched to capecitabine and irinotecan after PD. Surveillance imaging demonstrated increasing liver masses and therapy was changed to atezolizumab. CT scans (1.4 months post atezolizumab) revealed a 44% increase in the liver mass (Fig. 3d) and patient died 5.6 months from the initiation of atezolizumab.

### Case #5

A 49-year-old man with esophagus NEC metastatic to mediastinal lymph nodes received chemotherapy with etoposide/cisplatin and then concurrent chemoradiotherapy. Therapy was changed to PD-L1 inhibitor durvalumab and CTLA-4 inhibitor tremelimumab after PD. One month later he had an incomplete bowel obstruction which prompted the physician to obtain imaging beforehand. Scans (1.2 months post durvalumab and tremelimumab) showed new lung, liver, T10–12 and L2–4 metastases (538% increase from baseline imaging) (Fig. 3e) which resulted in backache and paralysis of lower limbs. He died 3.8 months from starting durvalumab and tremelimumab.

## Discussion

As hyperprogression has been reported for a limited time, there is no uniform definition of hyperprogression as mentioned above. Firstly, the evaluation criteria for hyperprogression are different. Nowadays RECIST 1.1 [11] is widely applied in solid tumor evaluation. However, limitations exist when considering immunotherapy. ICIs could impact host antitumor response and may require additional time to achieve measurable or sustained clinical effects compared with traditional cytotoxic chemotherapy [12]. Immune-related response patterns have been observed in clinical trials, including increased size of tumor lesions or development of new lesions which are inflammatory cell infiltrates of immune cells or necrosis with subsequent decreased tumor burden [6, 7]. These pseudoprogression would have been classified prematurely as PD by WHO or RECIST 1.1 criteria. So the immune-related response criteria including irRC [7] and irRECIST [13] were published in 2009 and 2014. The core novelty of the irRC and irRECIST is the incorporation of measurable new lesions into total tumor burden and comparison of this variable to baseline measurements [12].

In addition, the evaluation criteria mentioned above are based on changes in tumor size at two time points but not take into account tumor growth dynamics. Fast-growing tumors are more likely to be classified as SD or PD even if the therapy has an antitumor activity, meanwhile slow-growing tumors are likely to be classified as SD even if there is none antitumor activity [14]. The researchers used different indicators including TGR, TGK and PP to evaluate tumor growth dynamics and the specific formulas are shown in Table 2. There is one question that if there is no measurable lesion before immunotherapy, TGR and PP would be meaningless and cannot be calculated. If these patients have a significant increased size of tumor lesions or new lesions after immunotherapy, they should be highly suspected as hyperprogression and then only the TGK could applicable.

**Table 2:** The related parameters of hyperprogression according to different researchers

	Champiat et al. [8]	Kato et al. [9]	Saada-Bouzid et al. [10]
Evaluation criteria	RECIST 1.1	irRC	RECIST 1.1 irRECIST
Tumor growth dynamics indicator	TGR	PP	TGK
Specific formula	TG = 3Log(S_T_ /S_0_) /(T-T_0_) TGR = 100(TG-1)	PP = (S_T_ -S_0_) /S_0_	TGK = (S_T_ -S_0_) /(T-T_0_)
Definition of hyperprogression	PD TGR_POST_ /TGR_PRE_ ≥ 2	TTF < 2 months S_POST_ /S_PRE_ ≥ 150% PP_POST_ /PP_PRE_ > 2	TGK_POST_ /TGK_PRE_ ≥ 2

T and T_0_ stand for two time points respectively. S_T_ and S_0_ stand for the sum of tumor burden at T and T_0_ respectively. TGR_PRE_ stands for the TGR calculated between pre-baseline and baseline, TGR_POST_ stands for the TGR calculated between baseline and first evaluation imaging. And the other subscripts of “PRE” and “POST” have the similar meanings. Abbreviation: TGR, tumor growth rate; PP, progression pace; TGK, tumor growth kinetics; PD, progressed disease; TTF, time to treatment failure

All above we think irRC and irRECIST should be applied when evaluating immunotherapy efficacy. In immune-related PD (irPD) patients, whether they have clinical benefits should be considered to inform the possibility of pseudoprogression which would decide the subsequent therapy. In the real irPD patients, tumor growth dynamics indicators mentioned above should be used to confirm patients who have hyperprogression. Researchers generally take the indicators as a ≥ 2-flod increase compare with pre-immunotherapy as the definition of hyperprogression. However there is no uniform threshold so far and large sample of research is necessary to determine the appropriate threshold.

Although ICIs have demonstrated salutary antitumor effects, including long-term remissions, it cannot be denied immunotherapy will aggravate the condition in some patients. It is necessary to identify predictors of hyperprogression in order not to treat these patients who might be harmed by ICIs. There are now several biomarkers partially capable of predicting response: PD-L1 expression/amplification, high tumor mutational burden and mismatch repair gene defects [2, 4, 15–19]. However there is no explicit evidence whether these biomarkers could predict the occurrence of hyperprogression. In our study, 15 patients had the detection result of MMR and 13 patients had that of PD-L1, in these patients there is no significant relationship between hyperprogression with MMR or PD-L1 status.

Champiat et al. [8] found that hyperprogression was closely related to age, the median age of hyperprogression and non-hyperprogression patients was 66 and 55 years old respectively (*P* = 0.007). In elderly patients (≥65 years old) the incidence of hyperprogression was 19% while < 5% in < 65 years old patients (*P* = 0.018). In our study the same tendency appeared, the median age in hyperprogressors was 63 and that in non-hyperprogressors was 52 although there was no statistic difference. In elderly patients the function of immune cells, chemotaxis, phagocytosis and intracellular killing of pathogens would decrease [20], but the mechanism associating with hyperprogression is not clear. Saada-Bouzid et al. [10] found that in patients with R/M HNSCC hyperprogression significantly correlated with the presence of a regional recurrence (90% versus 37%, *P* = 0.008). Kato et al. [9] investigated potential genomic markers associated with hyperprogression after immunotherapy and the results showed that MDM2/MDM4 and EGFR alterations were correlated with TTF < 2 months (*P* = 0.001, *P* = 0.004). Four of 6 patients with MDM2/MDM4 amplification and 2 of 10 patients EGFR aberration had hyperprogression. Further research found that patients with hyperprogression were all treated with PD-1/PD-L1 inhibitor, not with CTLA-4 inhibitor. ICIs could elevate the level of interferon (IFN)-γ [21], which in turn activates JAK-STAT signaling [22] leading to an increase in interferon regulatory factor (IRF)-8 expression [23]. Then IRF-8 binds to the MDM2/MDM4 promoter inducing their expression [23, 24], which could inhibits the p53 tumor suppressor [25, 26]. And when in the presence of MDM2/MDM4 amplification, hyperprogression could occur [9]. All above is a hypothesis and the exact mechanism linking MDM2/MDM4 amplification and hyperprogression is unclear.

The phenomenon of hyperprogression suggests that in some patients ICIs may promote tumor proliferation instead of repressing growth. It has been confirmed that cell-intrinsic PD-1 receptor could lead to tumor growth in melanoma [27]. And the immune system could promote tumor cells progression and metastasis by inducing local inflammation, DNA damage, angiogenesis, and matrix degradation et al. [28–30]. In addition, ICIs may also result in the upregulation of alternative immune checkpoints [31], and the overall effect is uncertain. ICIs are very likely to promote tumor proliferation via regulating the immune system.

In this study, we explored hyperprogression after immunotherapy in patients with malignant tumors of digestive system. TGK was used to evaluate tumor growth dynamics and hyperprogression was observed in 20% (5/25) of evaluable patients or 11.1% (5/45) of all patients. Three of 5 were NECs and the other 2 were adenocarcinomas. As we all know, NECs in digestive system are a group of highly malignant neoplasms. Patients live a median of 4–15.6 months after their diagnosis [32] and without treatment survival is merely 1 month [33]. The histopathology of 3 patients were all poorly differentiated carcinomas with Ki-67 index of 25–50, 50 and 90%, respectively. The NECs grow with a high proliferation index, but the grow rate is further increased after immunotherapy. Whether patients with NECs are likely to have hyperprogression is uncertain due to the small size of our series.

Our study had its limitations as well. The number of evaluated patients was small which limited the identification of clinicopathological features of hyperprogression. We elaborated the phenomenon of hyperprogression after immunotherapy in patients with malignant tumors of digestive system preliminarily. And with clinical trials launching and ICIs coming into the market, more patients would have the opportunity to receive immunotherapy, we are largening the sample of patients. We analyzed the rate of change of tumor burden with CT scans and evaluation time in this study, furthermore radiomics analyze would be our next research direction.

## Conclusions

In summary, our study demonstrated that hyperprogression was observed in a fraction of patients with malignant tumors of digestive system treated with ICIs. The definition and predictors of hyperprogression have not evaluated accurately, further research involving more patients treated with ICIs are needed.

## Data Availability

The datasets used and/or analyzed during the current study are available from the corresponding author on reasonable request.

## Abbreviations

CTLA-4: Cytotoxic T lymphocyte associated antigen-4
ICIs: Immune checkpoint inhibitors
IRF: Interferon regulatory factor
irPD: immune-related PD
MSI-H: Microsatellite instability-high
NECs: Neuroendocrine carcinomas
PD-1: Programmed cell death-1
PD-L1: Programmed cell death ligand 1
PP: Progression pace
R/M HNSCC: Recurrent and/or metastatic head and neck squamous cell carcinoma
TGK: Tumor growth kinetics
TGKR: Tumor growth kinetics ratio
TGR: Tumor growth rate
TTF: Time to treatment failure
